# Seborrhoeic Keratosis of External Auditory Canal & its Management

**DOI:** 10.22038/IJORL.2023.67509.3307

**Published:** 2023-03

**Authors:** Anjan Kumar Sahoo, Namrata Chakraborty, Pavan Kumar Bonthu

**Affiliations:** 1 *Department of ENT and Head Neck Surgey, All India Institute oF Medical Sciences, BhopalMadhya Pradesh, India.*

**Keywords:** Ear, Recurrence, Seborrhoeic keratosis, Squamous cell carcinoma

## Abstract

**Introduction:**

Seborrhoeic keratosis (SK) is a benign neoplasm of the skin. They are usually found to occur anywhere in the body except palms, soles and mucous membranes. The skin of the external auditory canal is an extremely rare site for the occurrence of this benign neoplasm. Malignant transformation rarely occurs in this benign condition. It should be differentiated from other malignant condition like squamous cell carcinoma, basal cell carcinoma, Bowen’s disease, malignant melanoma or keratoacanthoma. Surgery is the mainstay of treatment though recurrence is very common. It can be removed by cryotherapy using liquid nitrogen or curettage, light fulgaration, shave removal or painting with pure TCA if the lesion is small. Diathermy shoul be used as minimal as possible to avoid scar formation.

**Case Report:**

An elderly female presented to ENT OPD with left ear blood-stained discharge. On inspection there was irregular blackish mass filling the entire left external auditory canal, fine needle aspiration cytology came to be seborrhoeic keratosis. Since on imaging the tumor was confined to the external auditory canal, it was excised completely by transcanal route. Surprisingly histopathology came to be squamous cell carcinoma. Considering the age and limited confinement of the tumor, she was kept on regular follow up.

**Conclusion:**

Seborrhoeic keratosis though a common benign tumor, malignant transformation may occur. Treatment is patient specific and may be modified considering the age and comorbidity of the patient.

## Introduction

Seborrhoeic keratosis (SK) is a benign neoplasm of the skin characterised by harmless skin lesions, mostly affect the elderly population (1) . There is a possibility of these lesions being associated with sun exposure and some predetermined genetic predisposition. They usually occur singly and sometimes in groups giving a characteristic stuck on appearance(2). They usually found to occur anywhere in the body except palms, soles and mucous membranes (3,4). 

The skin lining the external auditory canal is an extremely rare site for the occurrence of this benign neoplasm(1,4). Usually, seborrhoeic keratosis doesn’t require any treatment because of its benign nature and high chance of recurrence post excision. Surgery mostly required to rule out malignancy or for cosmetic purposes. Here we have described a case of seborrhoeic keratosis in the External auditory canal in an elderly female. This was very rare finding as there were only eighteen cases have been mentioned in literature (5). 

## Case Report

**Fig 1:**
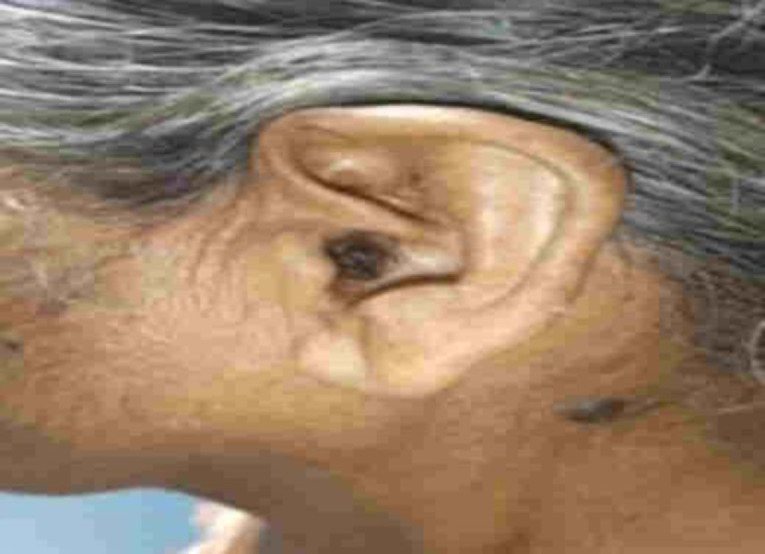
Lesion in the Left External auditory canal

A sixty four years old female presented to the END OPD with the complaints of left ear blood stained discharge, decreased hearing and ear pain for 30 days. She was hypertensive and on medication for last 10 years. On examination there was an irregular blackish mass was found filling the entire left EAC with blood clots along the floor (Figure 1). 

On probing the mass was found to be attached along the anterior and anterosuperior walls of the EAC. Tympanic membrane was not visualised. Rinne was negative on the pathological side with Weber being lateralised to left. Right EAC was clear with intact tympanic membrane and positive Rinne’s test. Pure tone audiogram revealed moderate conductive hearing loss with 56.67dBHL hearing loss in left ear and mild sensorineural hearing loss in the right ear with 30 dBHL hearing.

Fine Needle Aspiration Cytology was done which showed numerous mature squamous epithelial cells and anucleatesquames with no evidence of atypia or malignancy. Many horn cysts and squamous eddies were noted along with focal areas of basaloid proliferation and keratin pearl formation in the superficial layers with no dysplastic changes in the squamous epithelium. All these features were consistent with Seborrhoeic keratosis.

**Figure 2:**
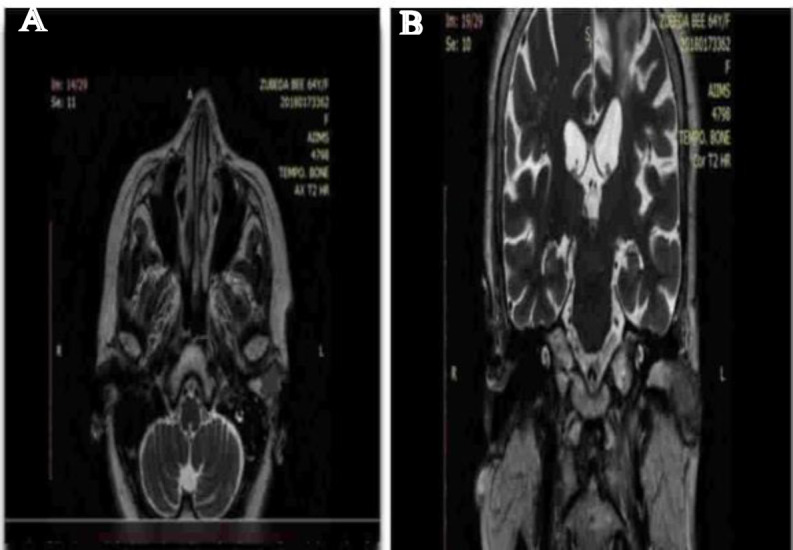
Magnetic Resonance Imaging of Temporal bone revealed a 1.5 x 2.9 x 1.2 cm sized lesion in the left EAC involving both the cartilaginous and bony part

Magnetic Resonance Imaging of Temporal bone revealed a 1.5 x 2.9 x 1.2 cm sized lesion in the left EAC involving both the cartilaginous and bony part which was isointense on T1 and hyper intense on T2 with diffusion restriction on DWI and intense post contrast enhancement (Figure 2). 

**Fig 3:**
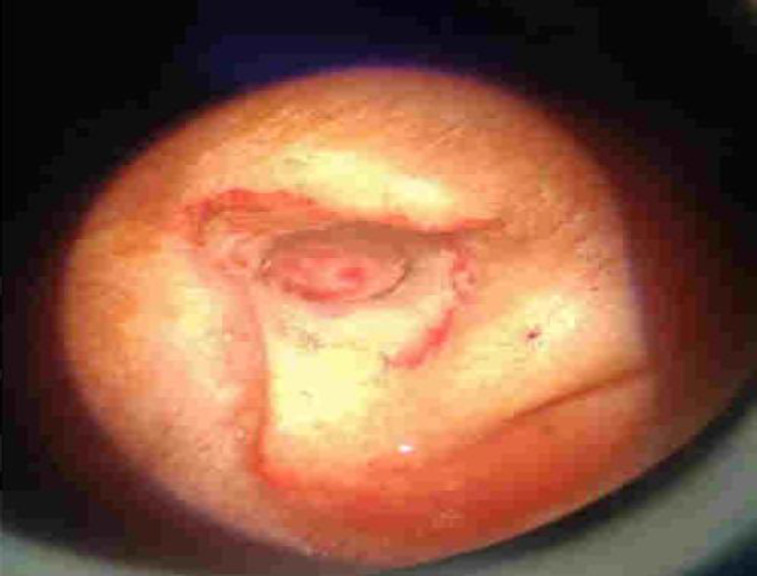
Normal tympanic membrane after excision of the mass

**Fig 4:**
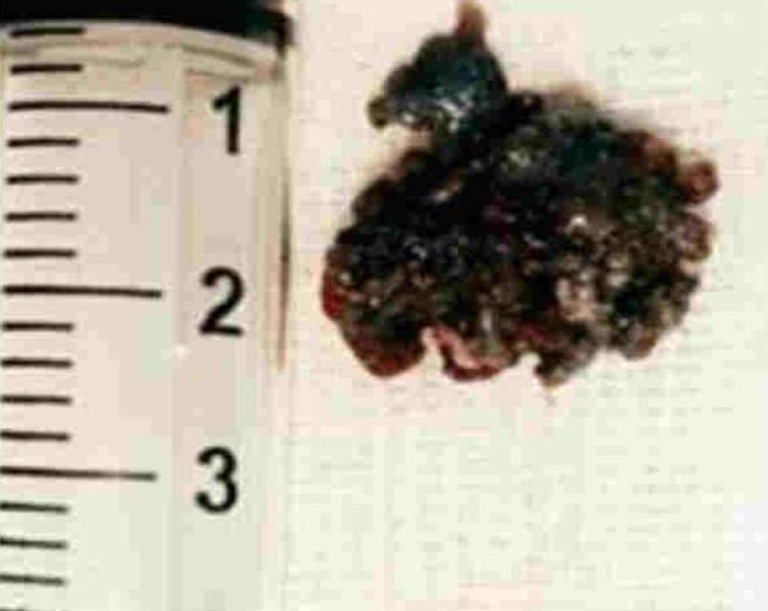
Mass after excision from the EAC

Anteriorly, the lesion was found to cause subtle erosion and thinning of the anterior wall of bony EAC with no extension into the TMJ. Anterolaterally it extends up to the tragus with no infiltration. Posteriorly, no extension was noted into the mastoid air cells. Medially, the lesion was found to cause mild concavity of the left TM. Medial edge of the lesion lies around 11.8 mm away from the TM. On corresponding CT cuts, no evidence of ossicular erosion was noted. Superiorly, no intracranial extension and inferiorly no parotid space invasion were seen. No significant lymph node enlargement was detected. The mass was excised and all the attachments were cauterised. Post excision tympanic membrane was found to be intact. (Figure 3 & 4). 

Post-surgery, patient improved symptomati- cally. Histopathology of the excised specimen revealed multiple tissue bits lined by hyperplastic stratified squamous epithelium giving rise to a lesion having shoulder and squamous cells, basaloid cells, horn cysts and pigment (seborrhoeic keratosis). At many foci, lesion gave rise to an invasive tumour arranged in nest sheets and intertwining islands. Tumour cells were polygonal to spindly, more hyperchromatic, show high N: C ratio and eosinophilic cytoplasm. Keratin formation and individual cell keratinisation were also seen. Dermis showed marked pigment incontinence and evidence of dermal infiltration. 

## Discussion

Seborrhoeic keratosis (SK) is one of the most common benign skin neoplasm (2). It is also known as basal cell papilloma/ senile warts/ brown warts/ seborrhoeic warts. They are most commonly seen in elderly population. There is equal predisposition in either of the sex groups. Africans or ethnicity with a darker skin colour have higher chance of developing them (3). 

SKs can occur on any part of the body, except palms, soles and mucous membrane. Shoulders, face, neck and chest were the most preferred site (3,4). EAC is a very rare site for seborrhoeic keratosis to develop and a total of 18 cases have been described in English literature (5). They were usually very small in diameter and occur due to prolonged exposure to ultraviolet radiation (6). The lesions usually begin with slight hyperpigmentation followed by light brown,waxy, flat scaly masses with a velvety to verrucous surface which gradually thicken to look like a wart (7) . With time, the colour darkens and becomes black, might be mistaken as melanoma. Sometimes, inflamed SKs might develop, where the lesions become swollen with erythematous base and might be confused with pyogenic granuloma or melanoma (3).

Histologically there occurs hyperkeratosis in between basal layer and keratinising surface of the epidermis (8) with variable melanocyte proliferation, elongation of dermal papillae, formation of keratin filled horn cysts and marked papillomatosis (2,3). 

Several histological variants have been identified which include: solid (most common), hyperkeratotic (rare), acanthotic, adenoid/ reticulated, clonal, irritated and melanoacanthoma (2,3,7). 

SKs mimic both malignant and premalignant lesions and might be very difficult to differentiate them clinically (4). Pigmented BCCs is also a possible differential but could be distinct out by their irregular with rolled edge and ulcerative centre (3). Actinic keratosis also occurs more on sun exposed areas but they are usually scaly, rough and erythematous. Viral warts are flesh coloured, usually found on palms and soles, with pin point haemorrhagic dots and are embedded in the skin. 

Though SKs are known to be benign, malignant association is also not uncommon. It can be associated with squamous cell carcinoma, basal cell carcinoma, Bowen’s disease, malignant melanoma or keratoacan-thoma (1). Our case it was associated with squamous cell carcinoma. SKs are removed either by cryotherapy using liquid nitrogen or curettage under LA (2,3), with diathermy to be used as minimal as possible to avoid scar formation. Other possible options include light fulgaration, shave removal or painting with pure TCA (2). Seborrhoeic keratosis has high chance of recurrence so it is better to counsel the patient to accept it rather than undergoing multiple treatments (3).

## Conclusion

Seborrhoeic keratosis is one of the most common benign skin neoplasms. But its occurrence in the skin of external auditory canal is very rare. Association with other malignant condition is the matter that was concerning to both the patients and doctors. 

Recurrence is very common. Early diagnosis and treatment with proper counselling to the patient will help the patient for proper management of the disease.
